# Single Molecule Investigation of Kinesin-1 Motility Using Engineered Microtubule Defects

**DOI:** 10.1038/srep44290

**Published:** 2017-03-13

**Authors:** Michael W. Gramlich, Leslie Conway, Winnie H. Liang, Joelle A. Labastide, Stephen J. King, Jing Xu, Jennifer L. Ross

**Affiliations:** 1 Department of Physics, University of Massachusetts Amherst, Amherst, MA 01003, USA; 2 Molecular and Cellular Biology Graduate Program, University of Massachusetts Amherst, Amherst, MA 01003, USA; 3 Department of Physics, University of California Merced, Merced, CA 95343, USA; 4 Burnett School of Biomedical Sciences, University of Central Florida, Orlando, FL 32827, USA

## Abstract

The structure of the microtubule is tightly regulated in cells via a number of microtubule associated proteins and enzymes. Microtubules accumulate structural defects during polymerization, and defect size can further increase under mechanical stresses. Intriguingly, microtubule defects have been shown to be targeted for removal via severing enzymes or self-repair. The cell’s control in defect removal suggests that defects can impact microtubule-based processes, including molecular motor-based intracellular transport. We previously demonstrated that microtubule defects influence cargo transport by multiple kinesin motors. However, mechanistic investigations of the observed effects remained challenging, since defects occur randomly during polymerization and are not directly observable in current motility assays. To overcome this challenge, we used end-to-end annealing to generate defects that are directly observable using standard epi-fluorescence microscopy. We demonstrate that the annealed sites recapitulate the effects of polymerization-derived defects on multiple-motor transport, and thus represent a simple and appropriate model for naturally-occurring defects. We found that single kinesins undergo premature dissociation, but not preferential pausing, at the annealed sites. Our findings provide the first mechanistic insight to how defects impact kinesin-based transport. Preferential dissociation on the single-molecule level has the potential to impair cargo delivery at locations of microtubule defect sites *in vivo*.

Molecular motor-based transport along microtubules is critical for the function and survival of all eukaryotic cells^1^,^2^,^3^. Disruptions to the transport process are linked to human diseases including neurodegeneration^4^,^5^. Toward understanding the mechanisms underlying transport disruption, prior studies have shed light on the effects of disease-relevant mutations^6^,^7^, co-factors^8^,^9^,^10^,^11^,^12^,^13^,^14^, microtubule-associated proteins^15^,^16^,^17^,^18^, and the biochemical nature of the microtubule tracks^19^,^20^ on motor functions. Despite these prior results, the effect of structural defects within a microtubule on motor-based transport has remained largely understudied.

Structural defects within microtubules represent discontinuities in the physical arrangement of tubulin dimers/subunits. Microtubule defects have long been observed in studies using cryo-electron microscopy^21^,^22^,^23^, scanning force microscopy^24^, and mechanical measurements of the microtubules^25^,^26^. Microtubules accumulate structural defects during polymerization, and the size of these defects can further increase when microtubules undergo mechanical stresses that are prevalent in living cells. Several cellular machineries are known to regulate microtubule structure *in vivo*. For instance, both the microtubule-associated proteins doublecortin and end binding 1 (EB1) preferentially stabilize microtubules with 13 protofilaments^23^,^27^, which can minimize defects associated with changes in protofilament number within individual microtubules. We and others have also shown that the microtubule severing enzyme, katanin, preferentially targets microtubule defects and, in turn, removes defects from the microtubule lattice^28^,^29^. The cell’s careful control in defect removal suggests that defects impact microtubule-based processes. This is corroborated by a recent study that sheds new light on the biological effect of defects on the dynamic instability of microtubule growth^30^. Here, we seek to understand the effect of microtubule defects on motor-based intracellular transport.

Because molecular motors must step along the microtubule to drive transport in cells, we hypothesized that microtubule defects likely disrupt motor-based transport. Supporting this postulation, we recently showed that microtubule defects alter the transport of cargos carried by multiple kinesins^31^. Interestingly, the effects depended on motor number: cargos driven by a few motors tended to dissociate prematurely from the microtubule, whereas cargos driven by more motors tended to pause. We speculated that different mechanisms underlie these different effects, with dissociations reflecting single-molecule effects and pauses arising from multiple-motor interactions^31^. However, direct testing of these potential mechanisms required investigations on the single molecule level, which has remained challenging.

An experimental difficulty of our prior work was that defects in microtubules occurred randomly during polymerization, and their locations could not be directly observed in motility assays. To bypass this issue, we previously used a modified optical trapping assay to repeatedly sample a single microtubule segment^31^, thereby inferring the presence of a defect based on the functional readout of cargo motility. However, as detailed below, our previous approach^31^ did not lend itself easily to mechanistic investigations on the single-molecule level. The reduced data throughput in single-molecule optical trapping assays^32^,^33^ limits the number of times a particular microtubule segment can be sampled in each experiment. The limited single kinesin processivity (~1 μm^32^,^33^,^34^) is also substantially smaller than the average spatial frequency of naturally-occurring defects (~6–17 μm depending on polymerization condition^22^), further reducing the probability of sampling a microtubule defect in each experiment.

In the current study, we annealed microtubules in end-to-end fashion^29^,^35^,^36^,^37^ to engineer defects that are directly observable using standard epi-fluorescence microscopy. We demonstrate that the annealed sites recapitulate the effects of naturally-occurring defects on multiple-motor transport uncovered in our previous study^31^. We next examined the effect of annealed microtubule defects on kinesin-1 motility at the single molecule level. We found that the defect sites promote the premature dissociation of kinesin from the microtubule, but did not appreciably influence the probability of single kinesins pausing during transport.

## Results and Discussion

### Engineering observable defects in microtubules via end-to-end annealing

**Figure 1:**
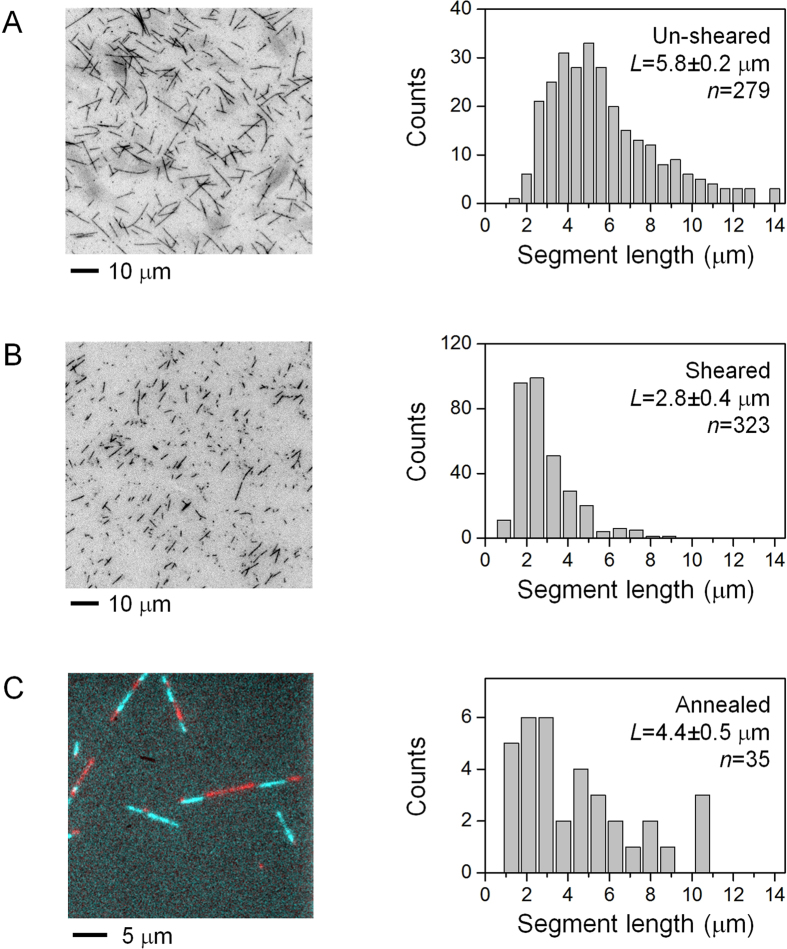
Representative fluorescence images and the corresponding distributions of the length of microtubules. *L* (±standard error) indicates the arithmetic mean and the associated error of microtubule lengths, and *n* indicates the sample size. (**A**) Un-sheared GMPCPP microtubules. (**B**) Sheared GMPCPP microtubules. (**C**) Annealed microtubules. Two populations of microtubules were differentially labeled prior to annealing. Cyan denotes GMPCPP microtubules, labeled with DyLight 488. Red denotes Taxol microtubules, labeled with DyLight 650. Distribution of segment length, quantified for GMPCPP segments (cyan).

We used end-to-end annealing^29^,^35^,^36^,^37^ to engineer defects that are directly observable during motility experiments (Fig. 1). Briefly, two populations of microtubules were polymerized *in vitro*, sheared to reduce their characteristic lengths (Fig. 1A,B), and then incubated together to induce end-to-end annealing (Fig. 1C). Differential fluorescence labeling of microtubules enables direct visualization of the annealed sites via standard epi-fluorescence microscopy (Fig. 1C). The presence of defects at these annealed sites has been verified previously via electron microscopy^35^.

We first characterized the effect of shearing on the average length of microtubule segments prior to annealing (Fig. 1A,B). For both the sheared and the un-sheared microtubules, we observed similar length distributions as in previous reports^36^,^38^ (Fig. 1A,B). Shearing halved the average length of the microtubule segment, from 5.8 μm un-sheared to 2.8 μm sheared under our experimental conditions (Fig. 1A,B). Shearing promotes end-to-end annealing of the microtubules^36^. The reduced length of the sheared microtubules increases the spatial frequency of annealed sites, and hence defects, within the annealed microtubule.

We next quantified the effect of annealing on the segmental length of annealed microtubules (Fig. 1C). We imaged individual segments based on their respective fluorescence labeling (red vs. cyan, Fig. 1C). We found that the average length of microtubule segments increased from 2.8 μm pre-annealing to 4.4 μm after annealing (Fig. 1B,C). The increase in length after annealing was expected, because, in order to use fluorescence to detect an annealed site, the site must contain a change in fluorescence labeling (interfacing cyan and red). However, during annealing, microtubules of the same color can also anneal together (cyan-to-cyan or red-to-red). Annealing of microtubules of the same color cannot be detected via fluorescence, leading to an increase in the apparent length of microtubule segments between the observable annealed sites. Importantly, we did not detect any decrease in microtubule segments length after annealing (Fig. 1C vs. B), indicating that the process of annealing did not reduce the length of the individual segments that are joined together.

Together, our data indicate that the distance between neighboring annealed sites (observable or not) is determined by the length of the sheared microtubule segments (Fig. 1B). We will use our characterization of sheared microtubules (Fig. 1B) to define a neighborhood surrounding an observable annealed site, such that the probability of encountering a non-observable annealed site in this neighborhood is minimized.

### Effect of annealed sites on few-kinesin transport recapitulates that of stochastic defects

**Figure 2:**
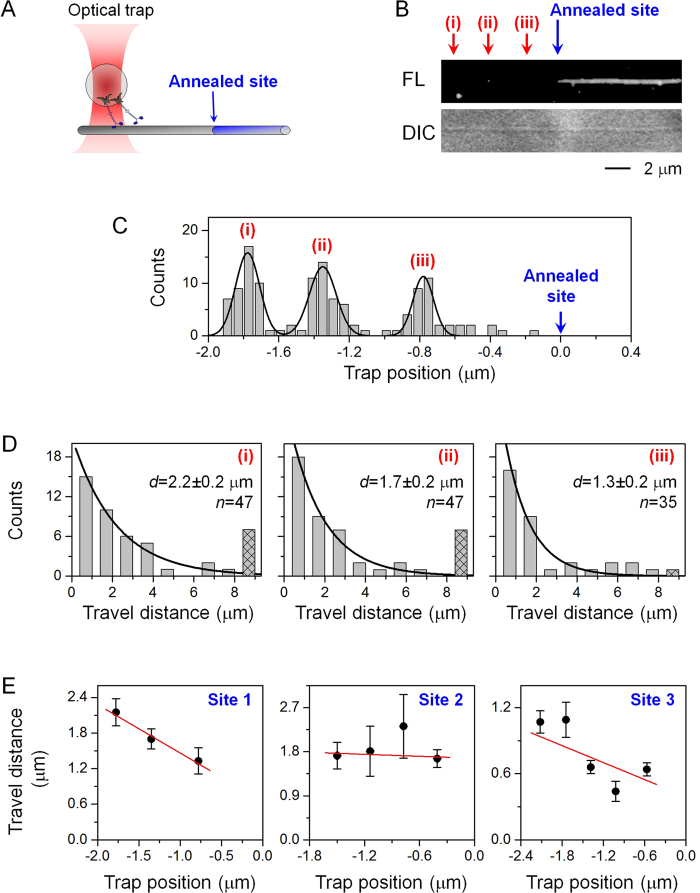
The effect of annealing on few-kinesin travel distance, examined using a range of optical trap positions for each annealed site (sites #1–3). (**A**) Schematic of optical trapping experiment. The position of the optical trap determines the starting position of kinesin-coated beads with respect to an annealed site. (**B**) Representative differential interference contrast (DIC) and epi-fluorescence (FL) images of an annealed microtubule. (i)-(iii) illustrate three distinct optical trap positions. (**C**) Distribution of initial starting positions of beads, as defined by three different optical trap positions relative to the annealed site. (**D**) Distribution of travel distances corresponding to each optical trap position, carried out in the same flow cell and using the same population of motor-coated beads. Hatched bar, cumulative counts of beads exceeding our field of view. Black line, best fit to a single exponential decay 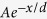. Mean travel distance (*d* ± standard error), and sample size (*n* trajectories) are indicated. (**E**) Mean travel distance as a function of optical trap position, measured for three different annealed sites. Error bars indicate standard error of the mean. The red line is a linear guide to the eye. Site 1 corresponds to the same annealed site examined in (**C**,**D**).

**Figure 3:**
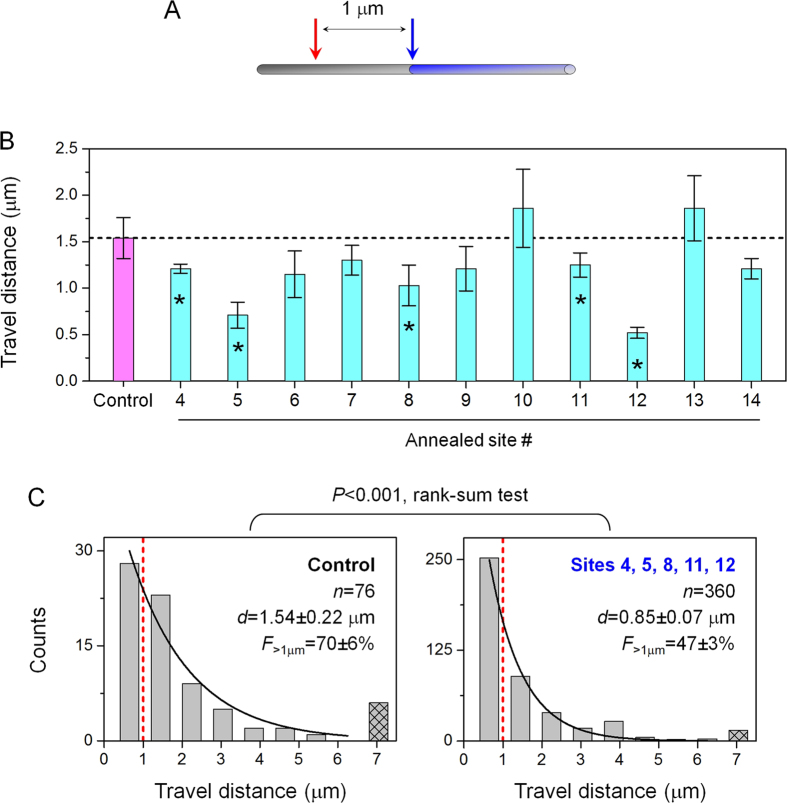
The effect of annealing on few-kinesin travel distance, examined using a fixed optical trap position for each annealed site (sites #4–14). (**A**) Experimental schematic. The position of the optical trap (red arrow) was held constant at 1 μm before each annealed site (blue arrow) and used to determine the starting position of few-motor cargos. (**B**) Mean travel distance of beads measured in the absence of an annealed site (Control, magenta) and in the presence of an annealed site (#4–14, cyan). Asterisks indicate *P* ≤ 0.058 when compared to Control (rank-sum test). Corresponding travel distributions are shown in Supplementary Information, Figs S1–S2. (**C**) Distributions of bead travel distances. Left panel indicates measurements in the absence of an annealed site (Control). Right panel indicates pooled distribution from sites that differed substantially from Control measurements (Sites 4, 5, 8, 11, 12). Hatched bar indicates cumulative counts of beads exceeding our field of view. Black solid line indicates best fit to a single exponential decay 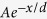. Red dashed line indicates 1 μm travel distance. Sample size (*n* trajectories), mean travel distance (*d* ± standard error), and the fraction of travel >1 μm (*F*_>1 μm_ ± standard error) are indicated. Standard error for *F*_>1 μm_ was determined as 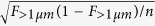.

We characterized the effect of annealed sites on the transport of cargos carried by a few kinesin motors (Figs 2 and 3), and compared the findings here to those from our prior work on naturally-occurring defects^31^. To directly compare the two studies, we used the same motor/bead ratio as in our prior study^33^, which resulted in a mean travel distance comparable to those reported for transport by exactly two kinesins (assembled using DNA/protein-based structures)^39^,^40^,^41^,^42^. We refer to these motor/bead complex as “few-kinesin cargos” because the number of motors decorating each bead is Poisson-distributed rather than well defined^32^,^33^. To increase the probability of defects at our annealed site, we mismatched the average number of protofilaments present in the two populations of microtubules that we annealed together (Type A annealed site, Table 1).

We first examined how the travel distance of few-kinesin cargos might vary as a function of distance they must travel before encountering an annealed site (Fig. 2). For each annealed site, we used a single-beam optical trap to position individual motor-coated beads to specific starting locations along the microtubule (Fig. 2A–C). For each starting position (as defined by the optical trap), we determined the distribution of bead travel distances under identical conditions (Fig. 2D,E). We then fitted each travel distribution to a single exponential decay (solid line, Fig. 2D) and determined the mean travel distance and the associated standard error as the fitted decay constant and associated uncertainty^32^. To reduce the likelihood of sampling a non-observable annealed site (between microtubules of the same fluorescence labeling), we limited the range of optical trap position to be within the characteristic length of the sheared microtubules (~2 μm in Fig. 2 vs. 2.8 μm in Fig. 1B). We anticipated that, the closer the starting position of cargos to the annealed site, the greater fraction of cargos will encounter a defect before stochastically dissociating from the microtubule, and the greater an impact the annealed site can have on cargo travel distance.

We measured the sensitivity of few-kinesin travel distance to the starting position of cargos along the microtubule (Fig. 2D,E). Because we sampled several starting positions for the same annealed site, and each experiment was limited by the duration of motor activity, we were limited in the sample size and thus the statistical power in this set of travel measurements (for example, *n* = 35–47 for Site 1, Fig. 2D). Despite this experimental limitation, for 2 of the 3 sites tested, the closer the starting position was to the annealed site, the smaller the apparent mean travel distance became (Sites 1 and 2, Fig. 2D,E). Comparing between the nearest and the furthest starting position, we detected a 38% reduction in travel distance for Site 1 (*P* = 0.158, rank-sum test) and a 40% reduction for Site 3 (*P* = 0.085, rank-sum test). Because travel measurements for each annealed site were carried out identically using the same bead preparation and in the same flow chamber, the key difference between them was their starting positions, or, the distance each bead can travel before it encounters the annealed site. Our data thus demonstrate that annealed sites represent a substantial barrier to the motility of few-kinesin cargos. This impaired travel distance effect of the annealed site (Fig. 2D,E) is consistent with our previous results for naturally-occurring defects^31^. Interestingly, for 1 of the 3 annealed sites tested, we did not detect any substantial effect of the annealed site on few-kinesin travel distance (Site 2, Fig. 2E). A possible explanation for this null effect is that, defects within this particular annealed site may not be large enough to substantially impact the motility of few-kinesin cargos. Additionally, a defect may be concealed by the interface between the microtubule and its support surface, and was not accessible to motors during transport. Importantly, we did not observe any evidence of an increased travel distance associated with the annealed site (Fig. 2D,E). This observation supports the current understanding that defects disrupt the microtubule track, and ought to impair motor-based transport.

We next examined an additional 11 annealed sites to understand the average impact of annealing on few-kinesin travel distance (Fig. 3B,C). Because of potential variations in annealed sites, it is formally possible that the effect observed for sites 1 and 3 (Fig. 2D,E) were atypically large for annealed sites. To test this possibility, we used the optical trap to initiate kinesin-coated cargos at a fixed, 1 μm distance before each annealed site (Fig. 3A). A fixed optical trap position enabled us to compare between different annealed sites identically, thereby addressing potential variations in annealing and/or the accessibility of individual sites. The 1 μm distance was chosen based on our data in Fig. 2: the effect of annealing on few-kinesin transport, when present, was readily observed at 1 μm distance (Sites 1 and 3, Fig. 2E). Further, the 1 μm distance is substantially shorter than the characteristic length of the sheared microtubule (2.8 μm, Fig. 1B), thus minimizing the probability of cargos encountering a non-observable annealed site during transport. To evaluate transport in the absence of any microtubule defects, we used un-sheared microtubules (without any annealed sites) and sampled bead travel distance among multiple microtubules (Control, Fig. 3B). This control measurement is minimally influenced by the presence of randomly occurring defects in the microtubule, because the probability that each trajectory encounters a polymerization-derived defect is low, and the locations of these naturally-occurring defects likely differ between microtubules.

We again found that annealing tended to impair the travel distance of few-kinesin cargos (Fig. 3B,C, S1-S2). For 5/11 annealed sites (45%), we measured a mean travel distance that was substantially smaller than the control value (*P* ≤ 0.058, rank-sum test, Fig. S1; asterisks, Fig. 3B). These data strongly support our finding in Fig. 2, and indicate that the impairment effect observed for sites 1 and 2 are not outliers. Combined, our data indicate a 50% probability for an annealed site to impair cargo transport by few kinesin motors (7/14 sites, Figs 2 and 3). We did not observe any instances where the mean travel distance, or the fraction of travel >1 μm, was substantially larger than the control value (*P* ≥ 0.31, rank-sum test; Fig. S2). When we pooled together measurements from the 5 sites that impaired bead transport, we uncovered a mean travel distance that was 45% smaller than the control value (*P* < 0.001, rank-sum test, Fig. 3C). The fraction of trajectories traversing beyond >1 μm (the location of the annealed site) also reduced by 33% from the Control case that lacked an annealed site (*F*_>1μm_, Fig. 3C). Thus, the impairment in bead travel distance was spatially correlated with the annealed site. At the same time, because 50% of trajectories traversed pass the annealed site (Fig. 3C, right), the defects that impair bead transport were likely local and did not span across all protofilaments at an annealed site.

Taken together, our data demonstrate that annealing impairs few-kinesin travel distance (Figs 2 and 3), recapitulating that of the naturally-occurring defects within the microtubule^31^. Thus, end-to-end annealing likely constitutes a simple and appropriate method for controllably generating defects with known locations that we can use in single molecule investigations.

### Single kinesins undergo premature dissociation at annealed sites

**Figure 4:**
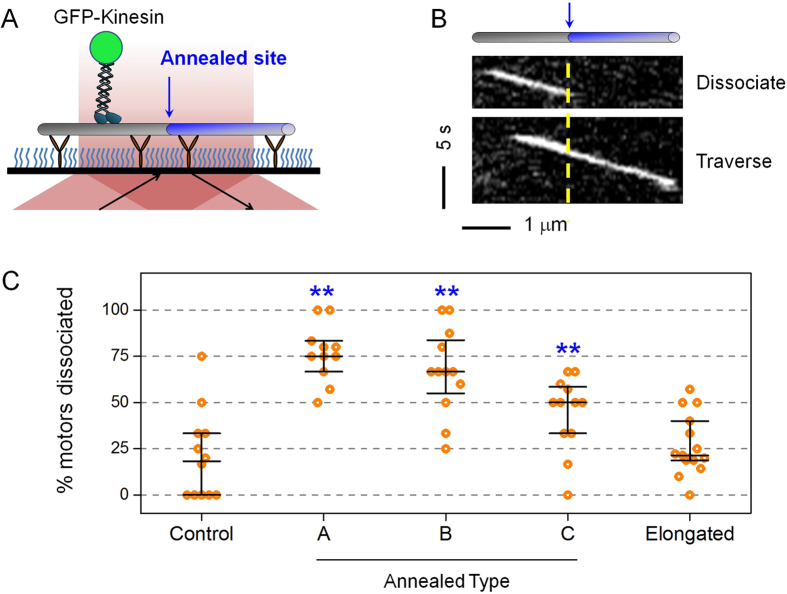
The impact of annealing on the probability of single kinesin dissociation from the microtubule. (**A**) Schematic of single molecule TIRF experiment. (**B**) Example kymographs representing the motility of single kinesin dissociating from (Dissociate) and traversing (Traverse) an annealed site (yellow dashed line and blue arrow). (**C**) The probability of single kinesin motor dissociating at a site on the microtubule. Control, positioned 0.675 μm away from an annealed site. Types A-C, annealed sites as described in Table 1. Elongated, junction between the GMPCPP seed and the elongation segment polymerized in the presence of Taxol. Horizontal bars indicate median values and quartiles. *n* = 12, 11, 12, 12, and 15 sites. 3–16 motors were tested for each site. Asterisks indicate sites that differ significantly from the Control site (*P* ≤ 0.021, rank-sum test). *P* values for comparisons between different sites are shown in Supplementary Information, Table S1.

We used a total internal reflection fluorescence (TIRF) assay to directly image the motility of single, GFP-labeled kinesins along annealed microtubules (Fig. 4A,B, Mov. S1–S2), and scored the probability of single kinesin dissociating at an annealed site versus elsewhere on the microtubule (Fig. 4C). Prior work has shown that kinesin-1 motors have difficulty navigating obstacles such as microtubule-associated proteins and other roadblocks^16^,^43^,^44^.

We first quantified the “baseline” probability of kinesin stochastically dissociating from the microtubule in the absence of defects (Control, Fig. 4C). For this control experiment, we examined a site that was positioned 0.675 μm away from an observable annealed site (Type A, characterized in Figs 2 and 3). This 0.675 μm distance is <1/4 of the average length of a sheared microtubule (2.8 μm, Fig. 1B) and is thus unlikely to contain any annealed sites that are not resolved via fluorescence imaging. This choice of control site also allowed us to contrast the motility of single kinesins between a control site and an annealed site under identical conditions, using the same motors and in the same flow chamber. Note that because this control site can still coincide with a naturally-occurring defect, the resulting analysis represents an upper-bound estimation for the true baseline value. We found that the baseline probability of kinesins detaching at the Control site was quite low. For 10 of the 12 sites tested (83%), the majority of the motors (>50%) that encountered a Control “site” were able to traverse the site (Control, Fig. 4C). Only a minority of the 48 motors tested (23 ± 6%) was observed to dissociate from the microtubule at a Control site.

We next used three different types of annealed sites to examine the effect of annealing on the probability of single kinesin dissociating from the microtubule. We generated these different types of defect sites by annealing together microtubules with different types of tubulin lattice structures^22^,^45^,^46^,^47^,^48^, as outlined in Table 1. For two of the three annealed types (Annealed Types A and B, Table 1), we mismatched the average number of protofilaments of the microtubules that we annealed together. We also generated a third type of annealed site with matching protofilament numbers (Annealed Type C, Table 1).

We found that annealing significantly increases the probability of single kinesins dissociation from the microtubule (Annealed Types A-C, Fig. 4C). For the majority of annealed sites tested ( ≥ 67%), we found that the majority of the motors ( ≥ 50%) that encountered an annealed site dissociated at the site (Fig. 4C). The fraction of motors that dissociated also doubled at an annealed site ( ≥ 47%, *n* = 50–58 motors), compared to the 23% observed for the Control site. These differences are statistically significant, as *P* ≤ 0.021 when we compared each Annealed Type with the Control site (rank-sum test, Table S1). We also detected significantly shorter travel distances for the sub-population of motors that dissociated at defects, versus those that traversed the annealed site, for all three annealed sites tested (Fig. S3). Taken together, our data indicate premature dissociation of the single kinesin motor from the microtubule at an annealed site.

We detected a significantly higher fraction of single kinesins dissociating at an annealed site with a protofilament mismatch than a site without a protofilament mismatch (Annealed Types A-B vs. Type C, Fig. 4C). Whereas ~70–77% of the motors tested dissociated at either a Type A or a Type B annealed site (*n* = 50–51 motors), only 47 ± 7% dissociated at a Type C annealed site (*n* = 58 motors). These differences are again statistically significant, as *P* ≤ 0.019 when we compared either Annealed Type A or B with Annealed Type C (rank-sum test, Table S1). This observation suggests that a mismatch in protofilament number increases the number and/or size of microtubule defects at the annealed site.

Interestingly, we did not detect a significant difference in single kinesin dissociating from a Type A versus Type B annealed site (Fig. 4C), even though we expected a larger degree of protofilament mismatch for the Type B annealed site based on prior studies^22^,^45^,^46^,^47^,^48^ (Table 1). The fraction of motors dissociated at the annealed site did not differ appreciably between the two annealed types (77 ± 6% for Type A, and 70 ± 7% for Type B, *n* = 50–51 motors). This difference is not statistically significant, as *P* = 0.291 when we compared between Annealed Type A and B (rank-sum test, Table S1). One possible explanation is that the effect of annealing arose from a change in the nucleotide state of the microtubule, rather than structural defects. This is a formal possibility, since both the Type A and the Type B sites interfaced between microtubule segments with different nucleotide states (GMPCPP and GDP, Table 1). To test this possibility, we polymerized GDP microtubules from GMPCPP seeds (“Elongated”, Fig. 4C), thus achieving the same change of nucleotide state as Type A and Type B annealed sites. At the same time, because microtubules can self-repair in the presence of free tubulin^26^, the Elongated site ought to contain substantially fewer structural defects than either the Type A or the Type B annealed sites. We found that the probability of single kinesins dissociating at the Elongated site (23 ± 4%, *n* = 129 motors) was significantly lower than the ~70–77% observed for either a Type A or a Type B annealed sites (*P* ≤ 0.025, rank-sum test, Table S1), but did not differ significantly from the 23% observed for the Control site (*P* = 0.338, rank-sum test, Table S1). Thus, a change in the nucleotide state of the microtubule was not sufficient to promote the premature dissociation of single kinesins from the microtubule. Our data here highlight the importance of structural defects on the effect of annealing on single kinesin function.

We speculate that, because end-to-end annealing is thermally driven, it may favor a lower degree of mismatch between the annealed microtubules, thereby minimizing the structural difference between a Type A and a Type B annealed site. Additionally, because microtubule self-repair at the Elongation sites mainly targets missing tubulin dimers^26^, the lack of effect at Elongation sites suggest that the defects impacting kinesin function likely correspond to missing tubulin dimers rather than gradual transitions in protofilament numbers. Interestingly, missing dimers can arise from bending and buckling microtubules^26^, which is frequently observed in microtubules in live cells^30^,^49^,^50^. Future investigations using cryo-electron microscopy^22^,^23^ could shed light on this exciting possibility and elucidate the structural details accompanying the functional impact of end-to-end annealing uncovered in the current study.

### Single kinesins do not pause preferentially at annealed sites

**Figure 5:**
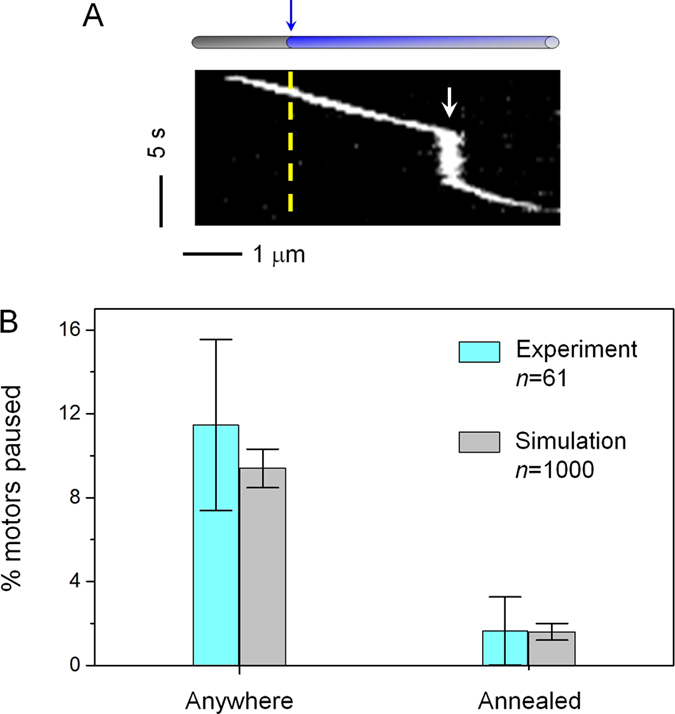
The impact of annealing on the probability of single kinesin pausing along the microtubule. (**A**) Example kymograph of a kinesin motor pausing along a microtubule (white arrow). Yellow dashed line and blue arrow indicate the location of the annealed site on the microtubule. (**B**) The probability of single kinesin pausing anywhere along the microtubule (Anywhere) versus at the annealed site (Annealed). Cyan indicates measurements from single molecule TIRF experiment. Grey indicates results of numerical simulation, assuming that kinesin does not pause preferentially at annealed sites (detailed in Methods). Error bars indicate the standard error for bionimal-distributed data, 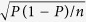, where *P* is the pausing probability and *n* is the sample size.

We next examined the effect of annealing on the probability of single kinesin pausing during transport (Fig. 5). We carried out this analysis using Anneal Type A, which demonstrated the most pronounced effect on single-kinesin dissociation probability (Fig. 4C). To minimize the probability of encountering an un-observed annealed site (between microtubules of the same color), we again limited our investigation to a well-defined, ±0.675-μm neighborhood surrounding each annealed site (as guided by Fig. 1B).

We found that only a limited fraction of motors exhibited pausing during transport (cyan, Fig. 5). Additionally, of the 61 motors that demonstrated motility within our neighborhood of interest, 7 motors (11.5 ± 4.1%) exhibited pausing (duration ≥3 s) anywhere along the microtubule, and only 1 pausing event (1.6 ± 1.6%) coincided at the annealed site. Thus, within the statistical power of our experiments, we did not detect any preference of single kinesin pausing at the annealed site. To examine this implication quantitatively, we carried out a numerical simulation with the assumption that the pausing of kinesin occurs randomly and does not differentiate between the annealed site or elsewhere along the microtubule (see Methods). We found that our experimental data are in excellent agreement with our numerical simulations (cyan and grey, Fig. 5). Together, our experimental data and simulation results strongly indicate that the kinesin motor does not pause preferentially at annealed sites. This finding is consistent with our previous finding that microtubule defects promoted cargo pausing only when the number of motors driving transport was large (~8 motors)^31^. The null-effect on single-kinesin level indicates that the pausing in many-motor cargos are due to inter-motor interactions, such as those that arise during a “traffic jam” between multiple motors at defects.

## Conclusions

In this study, we used end-to-end annealing to engineer microtubule defects that are directly observable using standard epi-fluorescence microscopy. Our data demonstrate that the impact of these annealed sites on few-kinesin transport recapitulated that previously reported for naturally-occurring defects. Thus, annealing offers a simple and appropriate experimental method to model microtubule defects. We found that single kinesins tended to dissociate prematurely at an annealed site, resulting in significantly impaired single kinesin processivity. Our findings disentangle the two modes of impact of microtubule defects (dissociating versus pausing) based on the number of motors engaged in cargo transport. Further, we showed that a change in the nucleotide state of tubulin at an annealed site was not responsible for these premature dissociation events. Instead, our data indicate that missing tubulin dimers, rather than gradual transitions in protofilament numbers, likely underlie the premature dissociation of single kinesins at microtubule defects. We did not detect any preference of single kinesins pausing at the annealed site. Our findings provide the first mechanistic insight to the impact of microtubule defects on multiple-motor-based cargo transport. Increased dissociation at the single molecule level would likely restrict the delivery of cargos or motors at microtubule defects in cells.

## Methods

### Protein purification

Tubulin protein used in TIRF studies was purified from pig brains as previously described^51^. Briefly, porcine brains were purchased from Adams Farm (Athol, MA) and homogenized at 4 °C. Debris were pelleted and removed, and soluble protein underwent two cycles of polymerization and de-polymerization with a final two cycles in the presence of high molar PIPES to remove microtubule-associated proteins. Purified tubulin was fluorescently labeled using a published protocol^52^, using either DyLight-650 or DyLight-488. Rhodamine-labeled tubulin and unlabeled tubulin protein used in optical trapping assays were purchased from Cytoskeleton (T240 and TL690M, Denver, CO).

Recombinant, truncated kinesin-1 motor protein used in TIRF studies was purified using bacterial expression as previously described^53^. Plasmid for the kinesin construct, pET17_K560_GFP_His, was purchased from Addgene (15219, Cambridge, MA). Kinesin and tubulin used in optical trapping studies was purified from cow brains as previously described^15^,^40^,^54^.

Ethics statement. This study does not report experiments on live vertebrates and/or higher invertebrates. No animals were sacrificed as part of this study.

### Microtubule preparation

Microtubules were polymerized *in vitro* and were free of microtubule-associated proteins. Three polymerization conditions: paclitaxel stabilized (Taxol microtubules), polymerized in 560 mM NaCl (High-salt microtubules), and polymerized with GMPCPP (GMPCPP microtubules) were used to alter the total number of protofilaments present in the resulting microtubules as previously described^22^,^46^,^47^,^48^.

Taxol-stabilized microtubules were polymerized in PEM-100 (100 mM PIPES, 2 mM EGTA, 1 mM MgSO_4_, pH 6.8) and in the presence of GTP. Tubulin (45 μM in PEM-100) was centrifuged at 300,000 × g at 4 °C for 10 min to remove aggregated tubulin in the pellet. 1 mM GTP was added to the recovered supernatant, and microtubules were polymerized at 37 °C for 20 min. Taxol was added at 20 μM final concentration to stabilize microtubules. The solution was then centrifuged at 16,000 × g for 10 min. The supernatant was removed, and the pellet was resuspended in PEM-100 with 20 μM Taxol and stored at room temperature.

High-salt microtubules were polymerized as described above for Taxol-stabilized microtubules, with the exception that PEM-100 was supplemented with 560 mM NaCl during microtubule polymerization.

GMPCPP microtubules were polymerized as described above for Taxol-stabilized microtubules, with the exception that GMPCPP was used in place of GTP as the nucleotide present during microtubule polymerization, and that the microtubules were polymerized at 37 °C for 1 hr.

Elongated microtubules were polymerized by including reduced concentration of tubulin (9 μM) with GTP to GMPCPP microtubule seeds, polymerized as above. These microtubules were allowed to polymerize for 20 microtubules at 37 °C. Microtubules were stabilized with 20 μM Taxol and centrifuged for 15 minutes at 8,000 × g in 25 °C to remove background tubulin. Microtubules were gently resuspended in PEM-100 with 20 μM Taxol. Microtubules were used within 5–6 hours of polymerizing to inhibit end-to-end annealing.

For single molecule total internal reflection fluorescence experiments, all types of microtubules, filaments were fluorescently labeled at a ratio of 1:20 labeled to unlabeled tubulin. Each type of microtubule was differentially labeled with rhodamine, DyLight-488, or Dylight-650 to distinguish the different segments. Microtubules were imaged using multicolor epi-fluorescence imaging. To generate annealed microtubules, a population of GMPCPP microtubules was sheared 4 times with a 50 μL Hamilton syringe and then incubated with a second, un-sheared microtubule population (Taxol microtubules, High-salt microtubules, or GMPCPP microtubules) in 1:1 ratio, at 37 °C overnight for end-to-end annealing.

For optical-trapping studies, microtubules were visualized using both epifluorescence microscopy and differential interference contrast (DIC) microscopy. Only Taxol microtubules were labeled fluorescently (1:3 labeled to un-labeled). The presence of rhodamine-labeled tubulin in microtubules had no significant impact on kinesin motility (data not shown). To generate annealed microtubules, 2 μM Taxol microtubules was mixed with 2 μM GMPCPP microtubules in equal volume, sheared with a 27.5 gauge needle 5 times, and then incubated at room temperature over night for end-to-end annealing.

### *In vitro* motility studies

Motility experiments were carried out in flow chambers *in vitro*. Flow chambers were constructed by sandwiching a coverslip (No. 1.5, Thermo Fisher Scientific, Waltham, MA) and a microscope slide (Thermo Fisher Scientific) using double-sided tape (Scotch 3 M, St. Paul, MN).

TIRF imaging was used to directly observe the motility of single GFP-tagged kinesins at annealed sites detected with epi-fluorescence in a separate color channel. The coverslip was silanized as previously described^34^,^55^,^56^. The flow chamber was incubated with 2% (v/v) anti-α-tubulin antibody (Sigma, St. Louis, MO) for 5 minutes. The remaining exposed surface was blocked with 5% Pluronic F127 (w/v) (Sigma, St. Louis, MO) for 5 minutes. Both the antibody and the Pluronic solutions were dissolved in PEM-20 (20 mM K-PIPES, pH 6.8, 2 mM MgSO_4_, 2 mM EGTA). Microtubules (900 nM) were introduced to the flow chamber and incubated for 10 minutes at room temperature. The flow chamber was washed using PEM-20, supplemented with 20 μM Taxol and 10 mM DTT. A motility mix containing 2–4 nM GFP-kinesin and ≥1 mM ATP was added to the chamber in imaging buffer (0.05% (w/v) Pluronic F127, 0.25 mg/ml BSA, 25 μM Taxol, 50 μM DTT, 0.5 mg/ml glucose, 0.5 mg/mL glucose oxidase, 0.15 mg/mL catalase in PEM-20). Experimental details of TIRF studies are as described previously^34^.

Optical trapping was used to quantify the motility of a polystyrene bead carried by a small group of kinesins at annealed sites. For optical trapping studies, the coverslip was plasma cleaned and then incubated with poly-L-lysine (0.00027% w/v in ethanol) for 10 minutes, and oven dried at 85 °C for 10 minutes. Microtubules (50 nM) were introduced to the flow chamber and incubated for 10 minutes at room temperature. The flow chamber was washed using PEM-20 supplemented with 1 mM GTP and 10 μM Taxol, and blocked with 5.55 mg/ml casein in PEM-20 (supplemented with 1 mM GTP and 10 μM Taxol). Before each set of optical trapping experiments, the location of the annealed site on a microtubule was visualized using epi-fluorescence and DIC, and the direction of bead travel was used to determine the structural polarity of the same microtubule. Experimental details of optical trapping studies are as described previously^31^. Importantly, we used a very weak trap power (<20 mW at fiber output^33^), such that the trap was sufficient to position individual beads but was not sufficient to stall beads carried by a single kinesin (stall force ~4.5 pN^11^). We also turned off the optical trap upon observation of directed bead motion along the microtubule, in order to enable bead transport in the absence of external load.

### Data analysis

Detailed quantifications for single-motor motility (TIRF studies) and for few-motor transport (optical trapping studies) are as described previously in refs 31 and 34, respectively. Position of motor, annealed site, and microtubules in TIRF experiments was digitized in 225-nm bins, which is the experimental resolution limit due to the combined effects of optical diffraction and thermal drift. Position of bead in Optical Trapping experiments was particle-tracked to 10 nm resolution using a template-matching algorithm as previously described^31^,^33^,^57^. To account for the human reaction time to manually shut off the optical trap, only trajectories >0.25 μm (whose transport persisted after the trap was turned off^31^,^32^,^33^) were used in analyses of bead travel distance in Figs 2 and 3.

### Simulation

We modeled the microtubule as a 1 × 7 lattice. The number of lattice sites were chosen to match both the length of the microtubule examined and the optical resolution of our TIRF experiments (a ± 0.675-μm neighborhood surrounding each annealed site, and 0.225 μm, respectively). Each simulation represented the pausing behavior of a single motor. Based on our experimental data that 7 of 61 motors paused anywhere along the microtubule, we constrained each simulation to have a 11.5% probability to exhibit pausing anywhere along the one-dimensional lattice. For each simulation (or, each simulated motor) that demonstrated a pausing event, we assumed that the motor has a uniform probability to pause at each of the seven simulated lattice site. For convention sake, we designated the simulation lattice site 4 as the annealed site. We repeated the simulation 1000 times to determine the probability of kinesin pausing at an annealed site (simulation lattice site 4) versus elsewhere along the microtubule (simulation lattice sites 1–3 and 5–6). We carried out the above simulation using a custom-routine in Matlab.

### Statistical analysis

The rank-sum test was used to determine the statistical difference between two sets of measurements in Figs 2, 3, 4, Figs S1–S3, and Table S1.

The standard error for the pausing probability was determined as 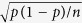, where *p* is the fraction of motile trajectories that paused, and *n* is the total number of motile trajectories observed. The standard error for the fraction of single kinesin dissociating at a site on the microtubule was determined as 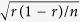, where *r* is the fraction of motors dissociated, and *n* is the total number of motors encountering the location.

## Additional Information

**How to cite this article:** Gramlich, M. W. *et al*. Single Molecule Investigation of Kinesin-1 Motility Using Engineered Microtubule Defects. *Sci. Rep.* **7**, 44290; doi: 10.1038/srep44290 (2017).

**Publisher's note:** Springer Nature remains neutral with regard to jurisdictional claims in published maps and institutional affiliations.

## Supplementary Material

Supplementary Information

Supplementary Movie 1

Supplementary Movie 2

## Figures and Tables

**Table 1:** Three types of annealed sites used in the current study.

Annealed Type	Microtubule 1	Microtubule 2	PF number at annealed site
A	Taxol (~12–13 PFs)	GMPCPP (14 PFs)	Mismatched
B	High-salt (~10–12 PFs)	GMPCPP (14 PFs)	Mismatched
C	GMPCPP (14 PFs)	GMPCPP (14 PFs)	Matched

When polymerized *in vitro*, nearly all of the GMPCPP microtubules (~95%) have 14 protofilaments^46^,^48^. Both the Taxol microtubules and the High-salt microtubules contain a mixed population of protofilaments^22^,^45^,^47^. PF, protofilament.
