# Supplementary material for: Single Molecule Investigation of Kinesin-1 Motility Using Engineered Microtubule Defects
Source: Sci Rep. 2017 Mar 13;7:44290. doi: 10.1038/srep44290 (PMC5347089; doi:10.1038/srep44290)
Supplement: Supplementary Information [file srep44290-s1.pdf]

SUPPLEMENTARY INFORMATION

Single Molecule Investigation of Kinesin-1 Motility Using Engineered Microtubule Defects

Michael W. Gramlich[1], Leslie Conway[2], Winnie H. Liang[3], Joelle A. Labastide[1], Stephen J. King[4], Jing Xu[3*], Jennifer L. Ross[1*]

[1.] Department of Physics, University of Massachusetts Amherst, Amherst, MA 01003

[2.] Molecular and Cellular Biology Graduate Program, University of Massachusetts Amherst, Amherst, MA 01003

[3.] Department of Physics, University of California Merced, Merced, CA 95343

[4.] Burnett School of Biomedical Sciences, University of Central Florida, Orlando, FL 32827

[*] Correspondence: Jing Xu (jxu8@ucmerced.edu), Jennifer L. Ross (rossj@physics.umass.edu)

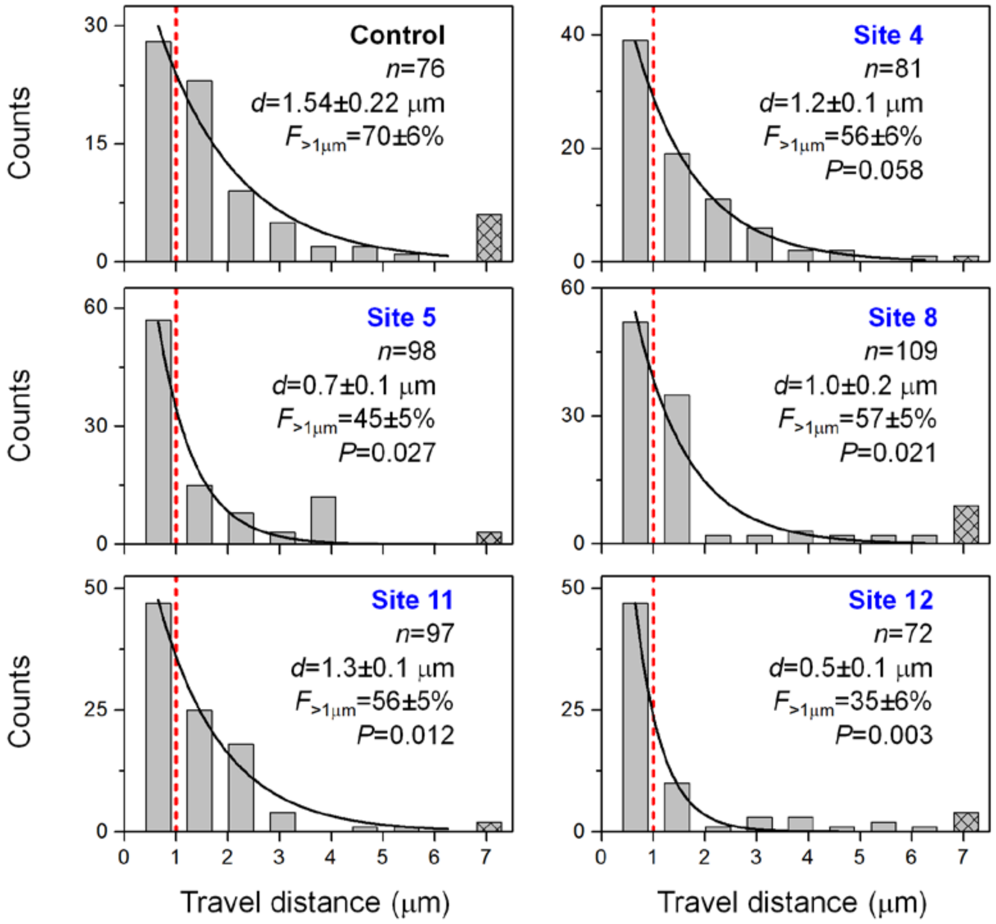

Figure S1. Distribution of travel distances for annealed sites that differed substantially from Control, corresponding to data shown in Figure 3B in the main text. Control indicates measurements in the absence of any annealed sites. Hatched bar indicates cumulative counts of beads exceeding our field of view. Black solid line indicates best fit to a single exponential decay $Ae^{-x/d}$. Red dashed line indicates 1 μm travel distance. Sample size (*n* trajectories), mean travel distance (*d* ± standard error), and the fraction of travel >1 μm ($F_{>1\mu m}$ ± standard error) are indicated. Standard error for $F_{>1\mu m}$ was determined as $\sqrt{F_{>1\mu m}(1-F_{>1\mu m})/n}$. *P*-value indicates comparison between each annealed site and Control, calculated using the rank-sum test.

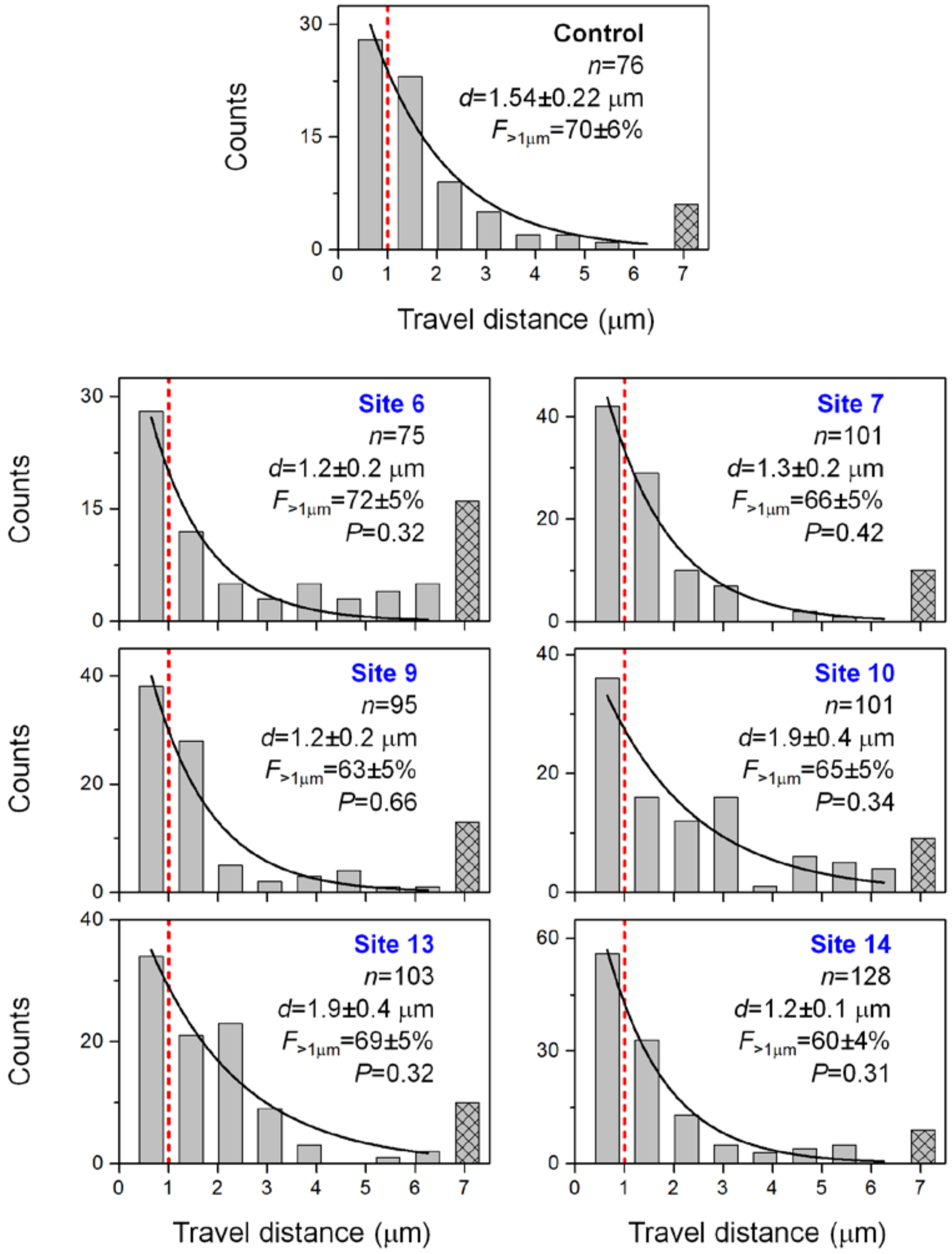

Figure S2. Distribution of travel distances for annealed sites that did not differ from Control, corresponding to data shown in Figure 3B in the main text. Control indicates measurements in the absence of any annealed sites. Hatched bar indicates cumulative counts of beads exceeding our field of view. Black solid line indicates best fit to a single exponential decay $Ae^{-x/d}$. Red dashed line indicates 1 μm travel distance. Sample size (*n* trajectories), mean travel distance (*d* ± standard error), and the fraction of travel >1 μm ($F_{>1\mu m}$ ± standard error) are indicated. Standard error for $F_{>1\mu m}$ was determined as $\sqrt{F_{>1\mu m}(1-F_{>1\mu m})/n}$. *P*-value indicates comparison between each annealed site and Control, calculated using the rank-sum test.

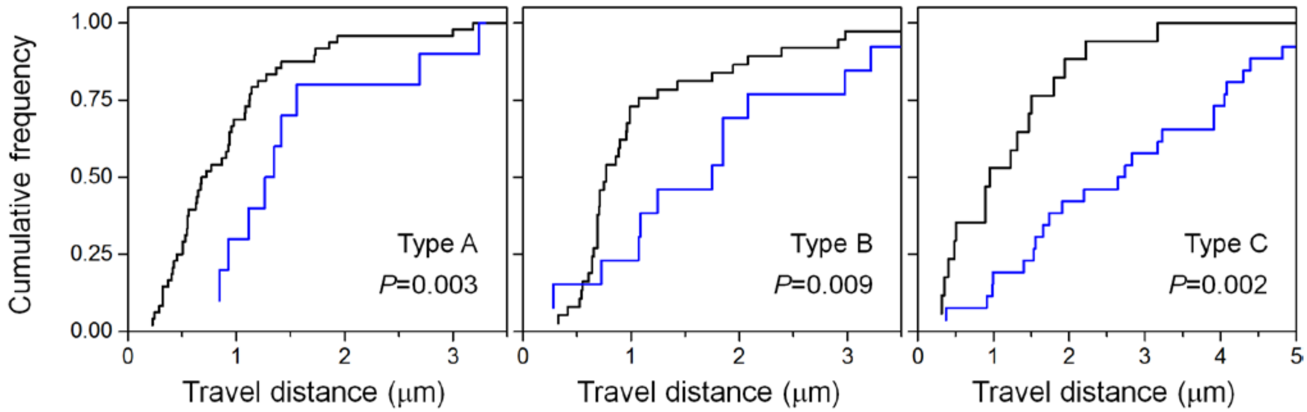

Figure S3. Cumulative frequency distributions of travel distance for single kinesin motors, measured for three types of annealed sites in TIRF experiment. Black line indicates the distribution of motors dissociating at an annealed site. Blue line indicates the distribution of motors traversing an annealed site. *P*-value indicates comparison between the motors that dissociated versus traversed each type of annealed site, calculated using the rank-sum test. The number of motors dissocated dissocated (or traversed) each type of annealed site is 48 (or 10) for Annealed Type A, 37 (or 13) for Anneald Type B, and 17 (or 26) for Annealed Type C. We observed significantly shorter single-kinesin travel distance for the motors that dissociated versus traversed the annealed site ($P \leq 0.009$, rank-sum test).

Comparison		*P*-value (rank-sum test)
Annealed Type A	Control	1.7×10^{-4}
Annealed Type B	Control	8.2×10^{-4}
Annealed Type C	Control	0.021
Annealed Type A	Annealed Type C	6.4×10^{-4}
Annealed Type B	Annealed Type C	0.019
Annealed Type A	Annealed Type B	0.291
Elongated	Annealed Type A	3.5×10^{-5}
Elongated	Annealed Type B	1.5×10^{-4}
Elongated	Annealed Type C	0.025
Elongated	Control	0.338

Tables S1. Statistical comparison for the probability of single kinesin dissociation from the microtubule at different types of sites shown in Figure 4 of the main text. In the absence of any functionally-relevant defect, the motor experiences a constant probability of dissociation at each step[1,2], and the probability distribution of individual kinesin dissociating from a particular location on the microtubule is well-described as a single-exponential decay[3]. We thus determined the *P*-values using the rank-sum test.

Movies S1-S2. Representative raw image sequences of a single kinesin motor traversing across (Movie S1) and dissociating at (Movie S2) an annealed site. Blue, single kinesin labeled with GFP. Green, microtubule labeled with Dylite 488 (in Movie S1) or rhodamine (in Movie S2). Red, microtubule labeled with DyLite 649. Arrows indicate the motility of each motor. Scale bar, 1 μm.

Supporting Reference

1 Seitz, A. & Surrey, T. Processive movement of single kinesins on crowded microtubules visualized using quantum dots. *EMBO J* **25**, 267-277 (2006).

2 Xu, J., Shu, Z., King, S. J. & Gross, S. P. Tuning multiple motor travel via single motor velocity. *Traffic* **13**, 1198-1205 (2012).

3 Block, S. M., Goldstein, L. S. & Schnapp, B. J. Bead movement by single kinesin molecules studied with optical tweezers. *Nature* **348**, 348-352 (1990).
